# Sex differences in the association between adipose insulin resistance and non-alcoholic fatty liver disease in Chinese adults

**DOI:** 10.1186/s13293-023-00549-0

**Published:** 2023-10-09

**Authors:** Ying Wei, Jia Liu, Guang Wang, Ying Wang

**Affiliations:** 1 Department of Endocrinology, Beijing Chao-Yang Hospital, Capital Medical University, No. 8, Gongti South Road, Chaoyang District, Beijing, 100020 China; 2 Health Management Center, Beijing Chao-Yang Hospital, Capital Medical University, Beijing, China

**Keywords:** Sex, Non-alcoholic fatty liver disease, Adipose tissue, Insulin resistance, Hyperlipidemias, Menopause, Age

## Abstract

**Background:**

Adipose insulin resistance (Adipo-IR) is associated with multiple metabolic diseases, including non-alcoholic fatty liver disease (NAFLD). The study aimed to evaluate sex differences in the association between Adipo-IR and NAFLD, and further investigated other potential modifiers.

**Methods:**

This cross-sectional study enrolled adults without diabetes who underwent physical examinations in Beijing Chao-Yang Hospital. We calculated the Adipo-IR index as the product of the fasting insulin and free fatty acid concentration. We categorized Adipo-IR into four groups according to quartiles, using the first interquartile range (Q1) as the reference. Logistic regression was used stratified by the modifiers after adjustment for potential confounders.

**Results:**

There were 5586 participants in the study, 49.8% (*n* = 2781) of whom were women and 30.4% (*n* = 1698) with NAFLD. There was a graded positive association between Adipo-IR and NAFLD, with sex (*P* = 0.01) and hyperlipidemia (*P* = 0.02) modifying this association. In the hyperlipidemic women, for one unit increase in log-Adipo-IR, the odds of having NAFLD increased by 385% after adjustment for potential confounders (OR = 4.85, 95%CI 3.54–6.73, *P* < 0.001). However, it turned out that the odds of having NAFLD increased by 131% (OR = 2.31, 95%CI 1.74–3.11, *P* < 0.001), 216% (OR = 3.16, 95%CI 2.56–3.93, *P* < 0.001), 181% (OR = 2.81, 95%CI 1.88–4.28, *P* < 0.001) in normolipidemic men, hyperlipidemic men, and normolipidemic women, respectively. Similarly, the ORs for the association between Adipo-IR and NAFLD in women with age ≥ 50 years were higher than ORs in women with age < 50 years.

**Conclusions:**

The positive correlation between Adipo-IR and NAFLD was stronger in hyperlipidemic women, compared with normolipidemic or hyperlipidemic men, or normolipidemic women. The association also strengthened for women over 50 years. Treatment strategies targeting Adipo-IR to alleviate NAFLD may be of value, especially in hyperlipidemic women after menopause.

## Plain language summary

In our study, we focused on non-alcoholic fatty liver disease (NAFLD), a condition characterized by excessive fat in the liver not caused by alcohol use. NAFLD is often connected to problems with how our body uses insulin, a hormone that helps regulate sugar levels. Adipose insulin resistance (Adipo-IR), or the inability of adipose cells to respond properly to insulin, can lead to more fat getting stored in the liver, worsening NAFLD. Previous research has indicated that NAFLD risk factors and outcomes can vary between sexes. For example, men are generally more susceptible to NAFLD during their reproductive years, whereas postmenopausal women have a risk level similar to or even exceeding that of men. We were particularly interested in how Adipo-IR affects the risk and progression of NAFLD differently in men and women. We conducted a large-scale study involving over 5,000 Chinese adults without diabetes. Our findings reveal a stronger association between Adipo-IR and NAFLD in women, especially in those with high lipid levels in their blood or who are over 50 years. This implies that treatments targeting Adipo-IR, such as weight loss strategies and certain medications, such as thiazolidinediones, might be particularly effective for these groups of women. Given the rising global prevalence of NAFLD, our research offers valuable insights for tailoring treatment strategies and could help identify which populations would benefit most from targeted interventions to mitigate the disease.

## Highlights

- Adipo-IR is positively correlated with NAFLD in subjects without diabetes.
- The positive association between Adipo-IR and NAFLD is sex-specific, and it strengthened for hyperlipidemic women.
- Among all the lipid indicators, low-density lipoprotein cholesterol acted as the main modifier.
- The association also strengthened for women over 50 years compared with women less than 50 years.

## Introduction

Dysregulated lipid and fatty acid metabolism are closely related to glycometabolism. Impairment of insulin sensitivity of the adipose tissue, that is, adipose insulin resistance (Adipo-IR), inhibits insulin-dependent lipogenesis and anti-lipolysis, results in excess free fatty acid (FFA) delivery to other tissues, and aggravates ectopic fat deposition and insulin resistance (IR) in liver and muscle (lipotoxicity) [1]. Adipose tissue insulin resistance index (Adipo-IR index), which is calculated as the product of the fasting insulin and FFA concentration [2], has been shown to correlate with 50% suppression of lipolysis (IC50) using a multistep pancreatic clamp, a gold standard measure of adipose tissue insulin sensitivity [3]. This simplified index method is reliably quantified for Adipo-IR and is useful in large-scale clinical practice [4].

Adipo-IR has been shown to associate with multiple metabolic diseases, including diabetes [5–7], hypertension [8], hyperuricemia [9], aortic valve calcification [10], dyslipidemia, polycystic ovary syndrome [11] and non-alcoholic fatty liver disease (NAFLD) [12–15]. NAFLD is characterized by excessive fat deposition in the liver without excessive alcohol consumption, which has been considered the hepatic manifestation of metabolic syndrome [16]. Some previous research revealed that sex differences do exist in the prevalence, risk factors, fibrosis, and clinical outcomes of NAFLD, such as the prevalence of NAFLD being higher in men during the reproductive age but lower than or comparable with women after menopause [17, 18]. Another study also revealed the sex differences in Adipo-IR, showing that severely obese males had more severe Adipo-IR than similarly obese females. The sex difference was associated with serum testosterone levels, which might inhibit Adipo-IR in obese males but promote Adipo-IR in obese females [19]. The receptor for advanced glycation end products also displayed sex-specific differences in Adipo-IR, with different expressions of genes involved in anti-oxidant and browning and insulin-induced AKT phosphorylation [20].

Adipose tissue is a principal source of fatty acids for hepatic triglyceride synthesis [21], and Adipo-IR is considered a major contributor to NAFLD, through the induction of hepatic IR and muscle IR by “lipotoxicity” [13–15, 22]. In addition, the association between non-alcoholic steatohepatitis (NASH) and Adipo-IR was independent of the degree of obesity [2]. The study also showed that amelioration of Adipo-IR by pioglitazone was closely related to histological improvement in patients with NASH [2]. Although the association between Adipo-IR and NAFLD has been well-established, there have not been studies exploring sex differences or other potential modifiers on the relationship. With the rapidly rising incidence and prevalence of NAFLD worldwide [23], this interaction effect will greatly enhance our knowledge in understanding what specific population would benefit most from ameliorating Adipo-IR to alleviate NAFLD progression.

Therefore, in the study, we recruited a large group of population in China, evaluated sex differences on the effect of Adipo-IR on NAFLD, and further investigated other potential modifiers on the association between Adipo-IR index and NAFLD.

## Materials and methods

### Study population

Participants of the study were adults who underwent physical examinations between April 2016 and August 2021 in Beijing Chao-Yang Hospital. Since the use of insulin may affect the fasting insulin levels and the calculation of Adipo-IR and we did not have data about medications, we only recruited participants without diabetes. Subjects who met the following criteria were excluded: (1) missing data in fasting blood insulin, fasting FFA, or liver ultrasound; (2) age was less than 18 years; (3) previous history of diabetes; (4) severe renal or liver dysfunction (denoting as estimated glomerular filtration rate (eGFR) < 60 ml/min/1.73m^2^, alanine transaminase (ALT) or aspartate transaminase (AST) > 400U/L). At last, the study enrolled 5586 participants in analysis and got informed consent from all subjects. The study was approved by the Ethical Review Board at Beijing Chao-Yang Hospital (Approval number: 2022-Science-517).

### Measurement of clinical information

Medical information of all participants was regularly collected by qualified physicians, including age, sex, weight, height, and medical history. We collected venous blood samples from all participants in the morning after they had more than 8 h of overnight fasting, then measured some blood biochemical parameters by standard procedures in the clinical laboratories of Beijing Chao-Yang Hospital. These parameters were: AST, ALT, creatinine, total cholesterol (TC), triglyceride (TG), low-density lipoprotein cholesterol (LDL-C), high-density lipoprotein cholesterol (HDL-C), fasting blood glucose (FBG), fasting insulin, and FFA. We use glucose oxidase method to measure FBG, chemiluminescence method for fasting insulin, colorimetric enzymatic method for ALT, AST, creatinine, lipids, and FFA (Siemens Healthcare Diagnostics). We calculated body mass index (BMI) by a person’s weight in kilograms divided by the square of height in meters. Hypertension is diagnosed if the systolic blood pressure is ≥ 140 mmHg or the diastolic blood pressure is ≥ 90 mmHg measured on two different days [24]. Dyslipidemia was defined as TC ≥ 5.2 mmol/L, or TG ≥ 1.7 mmol/L, or LDL-C ≥ 3.4 mmol/L, or HDL-C < 1.0 mmol/L, or non-HDL-C ≥ 4.1 mmol/L [25]. According to the American Diabetes Association guidelines, the criteria for the diagnosis of diabetes were: fasting blood glucose ≥ 7.0 mmol/L or hemoglobin A1c (HbA1c) ≥ 6.5% (48 mmol/mol) or random plasma glucose ≥ 11.1 mmol/L with classic symptoms of hyperglycemia [26]. Based on the 2019 Guideline for the diagnosis and management of hyperuricemia and gout in China, serum uric acid concentrations greater than 420 μmol/L was defined as hyperuricemia, no matter in women or men [27]. eGFR was calculated by the Chronic Kidney Disease Epidemiology Collaboration (CKD–EPI) Eq. ^28^. Adipo-IR index was calculated as the product of the fasting insulin and FFA concentration (Adipo-IR index = fasting insulin (μIU/mL) × fasting FFA (mmol/L)) [2]. NAFLD was diagnosed by two experienced clinicians via liver ultrasound, and the diagnostic criteria were: bright hepatic echoes, increased hepatorenal echogenicity, vascular blurring of portal or hepatic vein, and subcutaneous tissue thickness, not related to excess alcohol use or other causes of liver disease [29].

### Statistical analysis

In the study, we described normal-distributed continuous variables as mean ± standard deviation (SD), non-normal distributed continuous variables as median (quartiles), and categorical variables as count (percentage) in Table 1. The Shapiro–Wilk test was performed to check normality. Non-normal distributed variables were log-transformed when they were put into models and when they were considered continuous for analysis (e.g., Adipo-IR index, BMI, FBG). We further categorized the Adipo-IR index into four groups according to quartiles in all participants, using the first interquartile range (Q1) as the reference group (Table 2). Interaction terms between the Adipo-IR index and other variables were added to the regression model to detect effect modification. We explored the association between Adipo-IR index and NAFLD by logistic regression models, stratified by sex and other modifiers after adjusting for potential confounders. We performed multiple imputations using chained equations (MICE) by the R package mice for missing data [30]. We imputed missing values for the following covariates: BMI (2.69%), and hypertension (1.54%). The type 1 error (α) for rejecting the null hypothesis was set at 0.05. Analyses were carried out using R version 4.1.2.

**Table 1:** Baseline information of the total subjects

	Men *n* = 2805 (50.2%)	Women *n* = 2781 (49.8%)	Overall (*n* = 5586)
Age (years)			
< 35	706 (25.2%)	917 (33.0%)	1623 (29.1%)
35 ≤ age < 45	772 (27.5%)	645 (23.2%)	1417 (25.4%)
45 ≤ age < 55	704 (25.1%)	580 (20.9%)	1284 (23.0%)
> 55	623 (22.2%)	639 (23.0%)	1262 (22.6%)
BMI (kg/m^2^)	25.4 [23.3, 27.6]	22.5 [20.4, 25.0]	24.1 [21.6, 26.6]
Hypertension (*n*,%)	578 (20.6%)	356 (12.8%)	934 (16.7%)
Hyperlipidemia (*n*,%)	1737 (61.9%)	1278 (46.0%)	3015 (54.0%)
Hyperuricemia (*n*,%)	1015 (36.2%)	78 (2.8%)	1093 (19.6%)
NAFLD (*n*,%)	1199 (42.7%)	499 (17.9%)	1698 (30.4%)
AST(U/L)	22 [20, 27]	20 [17, 23]	21 [18, 25]
ALT(U/L)	24 [18, 34]	15 [12, 20]	19 [14, 28]
eGFR (mL/min/1.73m^2^)			
≥ 90	2477 (88.3%)	2576 (92.6%)	5053 (90.5%)
60 ≤ eGFR < 90	328 (11.7%)	205 (7.4%)	533 (9.5%)
TC (mmol/L)	5.01 (0.91)	4.99 (0.96)	5.00 (0.93)
TC ≥ 5.2 mmol/L (*n*,%)	1099 (39.2%)	1050 (37.8%)	2149 (38.5%)
TG (mmol/L)	1.43 [1.02, 2.06]	1.03 [0.78, 1.43]	1.20 [0.87, 1.76]
TG ≥ 1.7 mmol/L (*n*,%)	1076 (38.4%)	459 (16.5%)	1535 (27.5%)
LDL-C(mmol/L)	3.12 (0.83)	2.90 (0.87)	3.01 (0.86)
LDL-C ≥ 3.4 mmol/L (*n*,%)	1002 (35.7%)	716 (25.7%)	1718 (30.8%)
HDL-C(mmol/L)	1.17 [1.00, 1.34]	1.50 [1.23, 1.70]	1.30 [1.10, 1.57]
HDL-C < 1.0 mmol/L (*n*,%)	495 (17.6%)	103 (3.7%)	598 (10.7%)
FBG (mmol/L)	4.93 (0.58)	4.78 (0.52)	4.85 (0.55)
Fasting insulin(μIU/mL)	8.20 [5.60, 11.50]	7.30 [5.30, 10.20]	7.70 [5.50, 10.80]
FFA (mmol/L)	0.46 [0.36, 0.59]	0.53 [0.40, 0.67]	0.50 [0.38, 0.63]
Adipo-IR (μIU/mL*mmol/L)	3.77 [2.36, 5.89]	3.80 [2.48, 5.76]	3.78 [2.43, 5.84]

*BMI* body mass index, *NAFLD* non-alcoholic fatty liver disease, *AST* aspartate transaminase, *ALT*: alanine transaminase, *eGFR* estimated glomerular filtration rate, *TC* total cholesterol, *TG* triglyceride, *LDL-C* low-density lipoprotein cholesterol, *HDL-C* high-density lipoprotein cholesterol, *FBG* fasting blood glucose, *FFA* free fatty acid, *Adipo-IR* adipose tissue insulin resistance index

**Table 2:** Univariate association between Adipo-IR and NAFLD stratified by sex

Adipo-IR quartiles	Men			Women		
	NAFLD	OR (95% CI)	*P*	NAFLD	OR (95% CI)	*P*
Q1 (*n* = 1397)	127/735	Ref.	–	26/662	Ref.	–
Q2 (*n* = 1396)	214/679	2.20 (1.72, 2.84)	< 0.001	60/717	2.23 (1.41, 3.64)	0.007
Q3 (*n* = 1400)	338/672	4.84 (3.81, 6.20)	< 0.001	127/728	5.17 (3.40, 8.16)	< 0.001
Q4 (*n* = 1393)	520/719	12.51 (9.76, 16.14)	< 0.001	286/674	18.03 (12.06, 28.08)	< 0.001

*Adipo-IR* adipose tissue insulin resistance index, *NAFLD* non-alcoholic fatty liver disease, *OR* odds ratio, *CI* confidence interval, *Ref.* reference

## Results

### Baseline information

As shown in Table 1, the study enrolled 5586 participants, 49.8% (n = 2781) of whom were women and 30.4% (*n* = 1698) with NAFLD. The average age was 43.8 years, with a median BMI of 24.1 kg/m^2^. The women group had a lower BMI (22.5 [20.4, 25.0] vs. 25.4 [23.3, 27.6] kg/m^2^), and a smaller proportion of hypertension (12.8% vs. 20.6%), hyperlipidemia (46.0% vs. 61.9%), hyperuricemia (2.8% vs. 36.2%), and NAFLD (17.9% vs. 42.7%) than men. The AST and ALT levels in the women were also lower than those of the men. Most (90.5%) of the subjects in the study were with normal renal function (eGFR ≥ 90 mL/min/1.73m^2^). Blood lipid levels were worse controlled in men, including higher levels of TG, LDL-C, and lower levels of HDL-C compared with the women, but there was no big difference in TC levels. The concentrations of FBG and fasting insulin were higher, and the FFA levels were lower in men. The Adipo-IR index did not differ much between men and women (3.77 [2.36, 5.89] vs. 3.80 [2.48, 5.76] μIU/mL*mmol/L).

### Univariate regression between Adipo-IR and NAFLD stratified by sex

We categorized the Adipo-IR index into four groups according to quartiles in all participants, using the first interquartile range (Q1) as the reference group (Table 2). The scale was as follows: Q1: 0.17 ≤ Adipo-IR ≤ 2.43, Q2: 2.43 < Adipo-IR ≤ 3.78, Q3: 3.78 < Adipo-IR ≤ 5.84, Q4: 5.84 < Adipo-IR ≤ 46.5 μIU/mL*mmol/L. 11.0%, 19.6%, 33.2%, and 57.9% of subjects in each interquartile range had NAFLD, respectively. We observed graded positive associations between Adipo-IR and NAFLD both in men and in women. In men, there were 2.20 (95%CI 95% CI 1.72–2.84, *P* < 0.001), 4.84 (95%CI 3.81–6.20, *P* < 0.001), and 12.51 (95%CI 9.76–16.14, *P* < 0.001) times higher odds of having NAFLD among subjects in the second lowest, the second highest, and the highest Adipo-IR quartile, respectively, compared to subjects in the lowest quartile of Adipo-IR. In women, the corresponding ORs seemed to be higher compared to ORs in men.

### Multiple regressions between Adipo-IR and NAFLD stratified by sex and hyperlipidemia/LDL-C

**Fig. 1:**
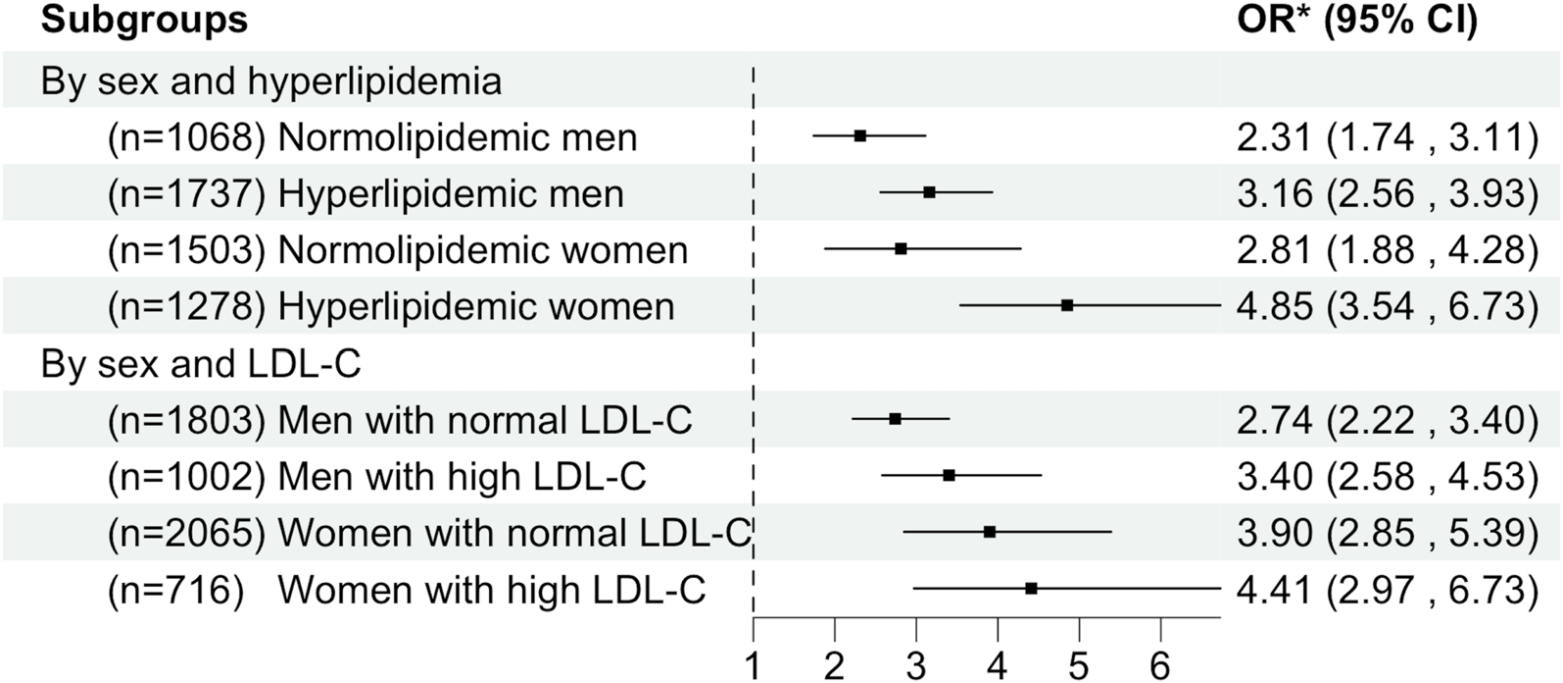
ORs between Adipo-IR and NAFLD stratified by sex and hyperlipidemia/LDL-C. *Adjusted for age, BMI, eGFR, FBG, hypertension, and hyperuricemia. All* P* values were < 0.001. Adipo-IR index, BMI and FBG were log-transformed when they were put into models. *OR* odds ratio, *CI* confidence interval, *LDL-C* low-density lipoprotein cholesterol, *Adipo-IR* adipose tissue insulin resistance index, *NAFLD* non-alcoholic fatty liver disease. Others were the same with Table 1

Hyperlipidemia (*P* = 0.02), and sex (*P* = 0.01) were significant modifiers in the association between Adipo-IR and NAFLD after adjusting for age, BMI, eGFR, FBG, hypertension, and hyperuricemia. Age, BMI, eGFR ≥ 90 mL/min/1.73m^2^, hypertension, and hyperuricemia were not significant modifiers (all *P* > 0.05). Therefore, we conducted subgroup analysis by sex and hyperlipidemia, as shown in Table 3. After adjusting for potential confounders, there was a graded positive association between Adipo-IR and NAFLD in each subgroup. However, the ORs for NAFLD in hyperlipidemic women were generally higher than ORs in normolipidemic or hyperlipidemic men, and normolipidemic women, indicating that the association between Adipo-IR and NAFLD was strongest in hyperlipidemic women. In the hyperlipidemic women, there were 1.63 (95%CI 0.82–3.39, *P* = 0.18), 3.63 (95%CI 1.92–7.36, *P* < 0.001), and 9.62 (95%CI 5.14–19.30, *P* < 0.001) times higher odds of NAFLD among subjects in the second lowest, the second highest, and the highest Adipo-IR quartiles, respectively, compared to subjects in the lowest quartile of Adipo-IR. Similarly, as shown in Fig. 1, in the hyperlipidemic women, for one unit increase in log-Adipo-IR, the odds of having NAFLD increased by 385% after adjustment for other potential confounders (OR = 4.85, 95%CI 3.54–6.73, *P* < 0.001). However, it turned out that the odds of having NAFLD increased by 131% (OR = 2.31, 95%CI 1.74–3.11, *P* < 0.001), 216% (OR = 3.16, 95%CI 2.56–3.93, *P* < 0.001), 181% (OR = 2.81, 95%CI 1.88–4.28, *P* < 0.001) in normolipidemic men, hyperlipidemic men, and normolipidemic women, respectively.

**Table 3:** Multiple regression between Adipo-IR and NAFLD stratified by sex and hyperlipidemia

Adipo-IR quartiles	Normolipidemic men (*n* = 1068)			Hyperlipidemic men (*n* = 1737)		
	NAFLD	OR (95% CI)^a^	*P*	NAFLD	OR (95% CI)^a^	*P*
Q1	54/405	Ref.	–	73/330	Ref.	–
Q2	62/285	1.30 (0.83, 2.06)	0.25	152/394	1.63 (1.15, 2.34)	0.007
Q3	84/216	2.27 (1.43, 3.61)	< 0.001	254/456	2.78 (1.97, 3.94)	< 0.001
Q4	94/162	4.17 (2.55, 6.88)	< 0.001	426/557	5.04 (3.53, 7.25)	< 0.001

Adipo-IR quartiles	Normolipidemic women (*n* = 1503)			Hyperlipidemic women (*n* = 1278)		
	NAFLD	OR (95% CI)^a^	*P*	NAFLD	OR (95% CI)^a^	*P*
Q1	13/432	Ref.	–	13/230	Ref.	–
Q2	18/402	1.35 (0.61, 3.07)	0.46	42/315	1.63 (0.82, 3.39)	0.18
Q3	46/393	1.99 (0.97, 4.28)	0.07	81/335	3.63 (1.92, 7.36)	< 0.001
Q4	67/276	3.73 (1.84, 7.96)	< 0.001	219/398	9.62 (5.14, 19.30)	< 0.001

*Adipo-IR* adipose tissue insulin resistance index, *NAFLD* non-alcoholic fatty liver disease, *OR* odds ratio, *CI* confidence interval, *Ref.* reference. Others were the same with Table 1

^a^Adjusted for age, BMI, eGFR, FBG, hypertension, and hyperuricemia

We further investigated which blood lipid components played an important role in this interactive effect, and we found that it was mainly LDL-C that modified the association between Adipo-IR and NAFLD (*P* = 0.02). We ran multiple regressions between Adipo-IR and NAFLD, stratifying by LDL-C (≥ 3.4 mmol/L) [25] and sex, adjusting for age, BMI, eGFR, FBG, hypertension, and hyperuricemia. Results in Fig. 1 also suggested Adipo-IR was a significant predictor of NAFLD in each subgroup. However, the association was strongest in women with high LDL-C, compared with women with normal LDL-C and men. In women with high LDL-C, one unit increase in log-Adipo-IR was associated with 4.41 times increase in odds for NAFLD after adjusting for other risk factors (OR = 4.41, 95%CI 2.97–6.73, *P* < 0.001).

### Multiple regressions between Adipo-IR and NAFLD in women stratified by hyperlipidemia and age

**Fig. 2:**
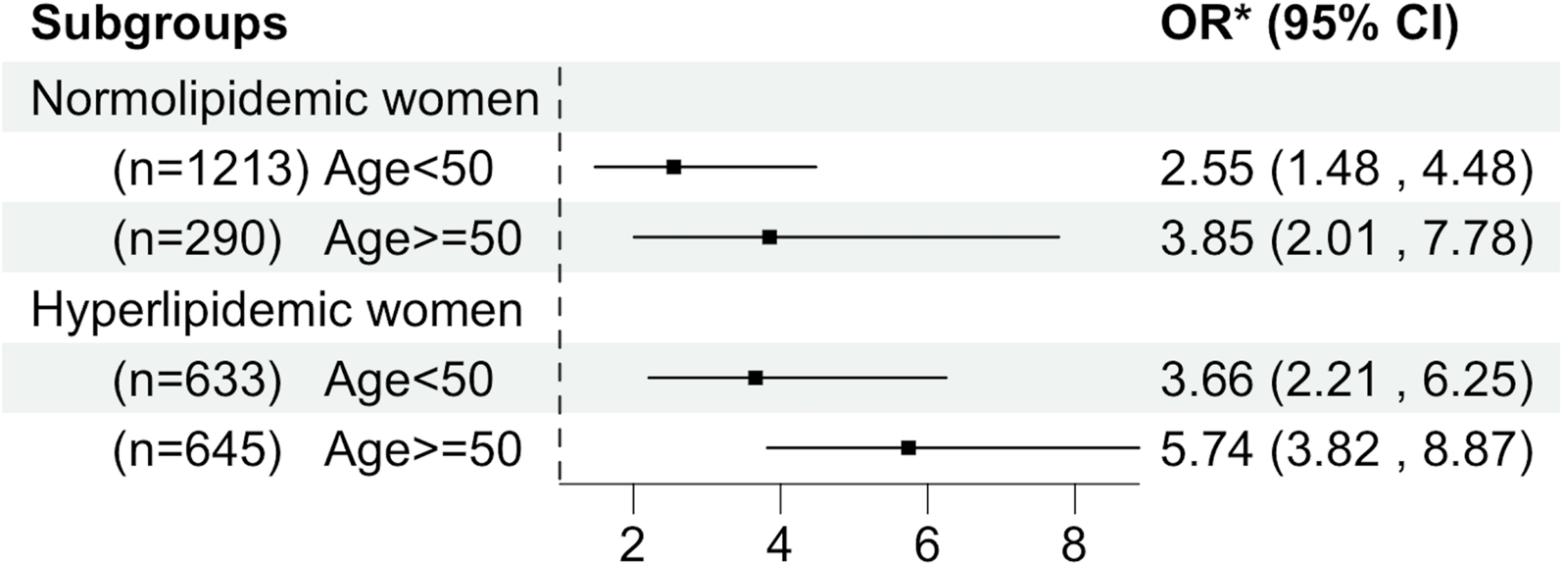
ORs between Adipo-IR and NAFLD in women stratified by hyperlipidemia and age. *Adjusted for age, BMI, eGFR, FBG, hypertension, and hyperuricemia. All* P* values were < 0.001. Adipo-IR index, BMI and FBG were log-transformed when they were put into models. *OR* odds ratio, *CI* confidence interval, *Adipo-IR* adipose tissue insulin resistance index, *NAFLD* non-alcoholic fatty liver disease. Others were the same with Table 1

Accounting for the effect of menopause, we further stratified women by hyperlipidemia and age (Fig. 2), although age was not a significant modifier in the relationship between Adipo-IR and NAFLD (*P* = 0.14). We found that the ORs in women with age ≥ 50 years were higher than ORs in women with age < 50 years, no matter in the normolipidemic or hyperlipidemic group, after adjusting for age, BMI, eGFR, FBG, hypertension, and hyperuricemia. Similar to previous results, the ORs in hyperlipidemic women were higher than ORs in normolipidemic women, no matter in the age < 50 or age ≥ 50-year-old group. On average, for one unit increase in log-Adipo-IR, the odds of having NAFLD increased by 474% (OR = 5.74, 95%CI 3.82–8.87, *P* < 0.001) in the hyperlipidemic women with age ≥ 50 years.

## Discussion

To sum up, our results showed a significant stepwise rise in the prevalence of NAFLD with the increase of Adipo-IR, after adjustment for several metabolic risk factors. This simplified index of Adipo-IR is a reliably quantified predictor for NAFLD in large-scale clinical practice. In addition, the positive association between Adipo-IR and NAFLD was stronger in hyperlipidemic women, especially in women with high LDL-C, compared with normolipidemic or hyperlipidemic men, or normolipidemic women. In the hyperlipidemic women, for one unit increase in log-Adipo-IR, the odds of having NAFLD increased 4.85-fold after adjustment for age, BMI, eGFR, FBG, hypertension, and hyperuricemia (OR = 4.85, 95%CI 3.54–6.73, *P* < 0.001). Furthermore, the association between Adipo-IR and NAFLD strengthened in women over 50 years compared with women less than 50 years.

Some previous research revealed that sex differences do exist in the prevalence, risk factors, fibrosis, and clinical outcomes of NAFLD [17]. However, data are very limited with respect to sex differences in the association between Adipo-IR and NAFLD. *Sun’s* research of 275 obese patients who underwent omental adipose tissue and liver biopsies showed that visceral adipocyte hypertrophy was associated with the onset and progression of NAFLD in females mediated by Adipo-IR. However, the association disappeared in males [31]. This result was consistent with our study showing that this relationship between Adipo-IR and NAFLD was reinforced in women. It could be speculated that females with severe Adipo-IR might require a more potent insulin sensitizer to improve glucose and lipid metabolism compared with males. One study also found that obese females in youth had higher Adipo-IR compared with obese males in youth, after adjustment for visceral adipose tissue, race, Tanner stage, and BMI [7]. Another study of obese adults indicated that males with class III obesity have more severe Adipo-IR than similarly obese females [19]. However, in our results, the Adipo-IR index did not differ significantly between men and women. The difference in the age and BMI of populations may account for the inconsistent results.

Interestingly, we found that the prevalence of NAFLD was lower in women compared to men (17.9% vs. 42.7%, *P* < 0.001). Nevertheless, in participants older than 55 years, the incidence was comparable between women and men (35.2% vs. 40.4%, *P* > 0.05), which aligns with the previous result that the prevalence of NAFLD is higher in men during the reproductive age but lower than or comparable with women after menopause, suggesting that estrogen is protective [17, 18]. Our results also indicated that the association between Adipo-IR and NAFLD strengthened in women after menopause. Studies suggested that estrogen could improve adipose tissue insulin sensitivity, and attenuate the lipolytic response through up-regulation of the number of antilipolytic α2A-adrenergic receptors [32, 33]. Although estrogen appears to have a protective effect on NAFLD, its role in lipid metabolism is complex. One study highlighted that estrogen-related receptor α, which acts downstream of estrogen/estrogen receptor α signaling, is a mediator modulating hepatic triglyceride-rich very low-density lipoprotein (VLDL-TG) assembly and secretion, which might contribute to the sex disparity in NAFLD development [34]. Interestingly, one previous study showed that testosterone had sex-specific and opposite effects on Adipo-IR; it was negatively correlated with Adipo-IR in overweight males but positively correlated with Adipo-IR in overweight females, suggesting low testosterone levels may contribute to more severe Adipo-IR in obese males [19]. Whether estrogen and testosterone mediate the sex difference in the association between Adipo-IR and NAFLD remains to be known.

Fat distribution may be another potential factor. Women accumulate more adipose tissue in the gluteo-femoral area than in the abdominal subcutaneous and visceral area compared with men [35], and the expandability and browning capacity of adipose tissue in women have been reported to be greater [18]. A previous study showed that abdominal obesity was strongly associated with Adipo-IR [36]. Adipose in the visceral area also has greater lipolytic rates and rates of proinflammatory adipokines production, exposing the liver to higher fatty acid concentrations [18]. Higher androgen levels in women facilitate abdominal obesity, while in men, androgen reduces abdominal obesity [37]. Therefore, any imbalance in gonadal hormone would influence adipose tissue distribution and function. Sex differences have also been reported for many adipokines, including leptin, adiponectin, chemerin, omentin, vaspin, lipocalin-2, glypican-4, and others [38], and some researchers speculated that estrogens might potentiate adiponectin sensitivity in liver [18]. In summary, sex and sex hormones are one of the greatest sources of biological heterogeneity in human diseases. Sex difference in the association between Adipo-IR and NAFLD deserves further investigation.

Our study further found that the association between Adipo-IR and NAFLD was reinforced in the presence of hyperlipidemia, especially high LDL-C. A study by Donnelly et al*.* indicated that around 60% of the fatty acids in triglycerides stored in the liver arise from adipose tissue [21]. FFAs and cholesterol accumulating in mitochondria could also lead to tumor necrosis factor alpha (TNFa)-mediated liver damage and reactive oxygen species (ROS) formation, acting as an early inflammatory hit leading to NAFLD pathologies [39–41]. Increased fatty acid uptake from the circulation and de novo hepatic lipogenesis (DNL), which saturates the capacity of the liver to oxidize fatty acids and secrete triglycerides in the form of very low-density lipoprotein (VLDL), are the main mechanisms of fatty liver [39, 42]. LDL-C is formed by selective removal of triglyceride from VLDL through catabolism. How LDL-C strengthens the relationship between Adipo-IR and NAFLD deserves further exploration. Similarly, previous research indicated that patients with NAFLD had deteriorated metabolic parameters, including higher plasma triglycerides and lower HDL-C compared to patients without steatosis [14]. Adipo-IR also correlated positively with TC, TG, LDL, and VLDL, and negatively with HDL [7]. The interaction between these different lipid metabolisms may exacerbate the development of NAFLD.

The study has several limitations. First, this is a single-center study in China only enrolling participants without diabetes. The results may not apply to populations from other countries or other ethnic backgrounds. Large cohorts with diabetes should be investigated to examine the interactive role of sex and hyperlipidemia on the association between Adipo-IR and NAFLD. The second limitation would be missing documentation of treatment modalities, such as physical lifestyle, which could act as a potential confounder of the association between Adipo-IR and NAFLD. Third, we do not have data about sex hormones, which might help us understand the hormonal effects and sex differences in the association between Adipo-IR and NAFLD. Finally, the retrospective and cross-sectional nature of the study limits the causal interpretation of our results.

## Conclusions

In summary, the current work expands our understanding about the role of Adipo-IR on NAFLD progression. Adipo-IR was a significant predictor of NAFLD in adults without diabetes after further adjustment for several metabolic risk factors. The incidence of NAFLD increased as Adipo-IR worsened. In addition, the positive association between Adipo-IR and NAFLD was stronger in hyperlipidemic women, especially in women with high LDL-C, compared with normolipidemic or hyperlipidemic men, or normolipidemic women. Furthermore, the association strengthened for women over 50 years compared with women less than 50 years.

## Perspectives and significance

Sex differences do exist in the association between Adipo-IR and NAFLD in adults without diabetes. Treatment strategies targeting Adipo-IR (e.g., weight loss and thiazolidinediones) to alleviate NAFLD may be of value, especially in hyperlipidemic women and women after menopause. Proper consideration of sex, age, blood lipids, and Adipo-IR will lead to a better understanding of NAFLD risk and therapeutic targets.

## Data Availability

The data are available from the corresponding author upon reasonable request.

## References

[1] Delarue J, Magnan C (2007). Free fatty acids and insulin resistance. Curr Opin Clin Nutr Metab Care.

[2] Gastaldelli A, Harrison SA, Belfort-Aguilar R (2009). Importance of changes in adipose tissue insulin resistance to histological response during thiazolidinedione treatment of patients with nonalcoholic steatohepatitis. Hepatology.

[3] Søndergaard E, Espinosa De Ycaza AE, Morgan-Bathke M, Jensen MD. How to Measure Adipose Tissue Insulin Sensitivity. J Clin Endocrinol Metab. 2017;102(4):1193–1199. 10.1210/jc.2017-00047 10.1210/jc.2017-00047 PMC5460729 28323973

[4] Ter Horst KW, van Galen KA, Gilijamse PW (2017). Methods for quantifying adipose tissue insulin resistance in overweight/obese humans. Int J Obes (Lond).

[5] Kotronen A, Juurinen L, Tiikkainen M, Vehkavaara S, Yki-Järvinen H (2008). Increased liver fat, impaired insulin clearance, and hepatic and adipose tissue insulin resistance in type 2 diabetes. Gastroenterology.

[6] Gastaldelli A, Gaggini M, DeFronzo R (2017). Role of adipose tissue insulin resistance in the natural history of type 2 diabetes: results from the San Antonio metabolism study. Diabetes.

[7] Kim J, Bacha F, Tfayli H, Michaliszyn S, Yousuf S, Arslanian S (2019). Adipose tissue insulin resistance in youth on the spectrum from normal weight to obese and from normal glucose tolerance to impaired glucose tolerance to type 2 diabetes. Diabetes Care.

[8] Sasaki N, Maeda R, Ozono R, Yoshimura K, Nakano Y, Higashi Y (2022). Adipose tissue insulin resistance predicts the incidence of hypertension: the Hiroshima Study on Glucose Metabolism and Cardiovascular Diseases. Hypertens Res.

[9] Sun H, Chang X, Bian N (2022). Adipose tissue insulin resistance is positively associated with serum uric acid levels and hyperuricemia in northern Chinese adults. Front Endocrinol.

[10] Jorge-Galarza E, Posadas-Romero C, Torres-Tamayo M (2016). Insulin resistance in adipose tissue but not in liver is associated with aortic valve calcification. Dis Markers.

[11] Mu L, Li R, Lai Y, Zhao Y, Qiao J (2019). Adipose insulin resistance is associated with cardiovascular risk factors in polycystic ovary syndrome. J Endocrinol Invest.

[12] Kalavalapalli S, Leiva EG, Lomonaco R (2022). Adipose tissue insulin resistance predicts the severity of liver fibrosis in patients with type 2 diabetes and NAFLD. J Clin Endocrinol Metab.

[13] Armstrong MJ, Hazlehurst JM, Hull D (2014). Abdominal subcutaneous adipose tissue insulin resistance and lipolysis in patients with non-alcoholic steatohepatitis. Diabetes Obes Metab.

[14] Lomonaco R, Ortiz-Lopez C, Orsak B (2012). Effect of adipose tissue insulin resistance on metabolic parameters and liver histology in obese patients with nonalcoholic fatty liver disease. Hepatology.

[15] Guerra S, Mocciaro G, Gastaldelli A (2022). Adipose tissue insulin resistance and lipidome alterations as the characterizing factors of non-alcoholic steatohepatitis. Eur J Clin Invest.

[16] Yki-Järvinen H (2014). Non-alcoholic fatty liver disease as a cause and a consequence of metabolic syndrome. Lancet Diabetes Endocrinol.

[17] Lonardo A, Nascimbeni F, Ballestri S (2019). Sex differences in nonalcoholic fatty liver disease: state of the art and identification of research gaps. Hepatology.

[18] Morán-Costoya A, Proenza AM, Gianotti M, Lladó I, Valle A (2021). Sex Differences in nonalcoholic fatty liver disease: estrogen influence on the liver-adipose tissue crosstalk. Antioxid Redox Signal.

[19] Li X, Liu J, Zhou B (2021). Sex differences in the effect of testosterone on adipose tissue insulin resistance from overweight to obese adults. J Clin Endocrinol Metab.

[20] Du Z, Wu J, Feng Z (2022). RAGE displays sex-specific differences in obesity-induced adipose tissue insulin resistance. Biol Sex Differ.

[21] Donnelly KL, Smith CI, Schwarzenberg SJ, Jessurun J, Boldt MD, Parks EJ (2005). Sources of fatty acids stored in liver and secreted via lipoproteins in patients with nonalcoholic fatty liver disease. J Clin Invest.

[22] Cusi K (2012). Role of obesity and lipotoxicity in the development of nonalcoholic steatohepatitis: pathophysiology and clinical implications. Gastroenterology.

[23] Huang D, El-Serag H, Loomba R (2021). Global epidemiology of NAFLD-related HCC: trends, predictions, risk factors and prevention. Nat Rev Gastroenterol Hepatol.

[24] Hypertension. World Health Organization. 25 August, 2021. https://www.who.int/news-room/fact-sheets/detail/hypertension. Accessed 1 Dec 2022.

[25] Joint committee for guideline revision (2018). 2016 Chinese guidelines for the management of dyslipidemia in adults. J Geriatr Cardiol.

[26] Classification and Diagnosis of Diabetes (2022). Standards of medical care in diabetes-2022. Diabetes Care.

[27] Chinese Society of Endocrinology, Chinese Medical Association (2020). Guideline for the diagnose and management of hyperuricemia and gout in China (2019). Chin J Endocrinol Metab.

[28] Levey AS, Stevens LA, Schmid CH (2009). A new equation to estimate glomerular filtration rate. Ann Intern Med.

[29] Khov N, Sharma A, Riley TR (2014). Bedside ultrasound in the diagnosis of nonalcoholic fatty liver disease. World J Gastroenterol.

[30] Azur MJ, Stuart EA, Frangakis C, Leaf PJ (2011). Multiple imputation by chained equations: what is it and how does it work?. Int J Methods Psychiatr Res.

[31] Sun H, Fang D, Wang H (2022). The association between visceral adipocyte hypertrophy and NAFLD in subjects with different degrees of adiposity. Hepatol Int.

[32] Pedersen SB, Kristensen K, Hermann PA, Katzenellenbogen JA, Richelsen B (2004). Estrogen controls lipolysis by up-regulating alpha2A-adrenergic receptors directly in human adipose tissue through the estrogen receptor alpha. Implications for the female fat distribution. J Clin Endocrinol Metab.

[33] Park YM, Pereira RI, Erickson CB, Swibas TA, Cox-York KA, Van Pelt RE (2017). Estradiol-mediated improvements in adipose tissue insulin sensitivity are related to the balance of adipose tissue estrogen receptor α and β in postmenopausal women. PLoS ONE.

[34] Yang M, Liu Q, Huang T (2020). Dysfunction of estrogen-related receptor alpha-dependent hepatic VLDL secretion contributes to sex disparity in NAFLD/NASH development. Theranostics.

[35] Goossens G, Jocken J, Blaak E (2021). Sexual dimorphism in cardiometabolic health: the role of adipose tissue, muscle and liver. Nat Rev Endocrinol.

[36] Jiang J, Cai X, Pan Y (2020). Relationship of obesity to adipose tissue insulin resistance. BMJ Open Diabetes Res Care.

[37] Escobar-Morreale HF, Alvarez-Blasco F, Botella-Carretero JI, Luque-Ramírez M (2014). The striking similarities in the metabolic associations of female androgen excess and male androgen deficiency. Human reproduction (Oxford, England).

[38] Valencak TG, Osterrieder A, Schulz TJ (2017). Sex matters: the effects of biological sex on adipose tissue biology and energy metabolism. Redox Biol.

[39] Tilg H, Moschen AR (2010). Evolution of inflammation in nonalcoholic fatty liver disease: the multiple parallel hits hypothesis. Hepatology.

[40] Feldstein AE, Werneburg NW, Canbay A (2004). Free fatty acids promote hepatic lipotoxicity by stimulating TNF-alpha expression via a lysosomal pathway. Hepatology.

[41] Marí M, Caballero F, Colell A (2006). Mitochondrial free cholesterol loading sensitizes to TNF- and Fas-mediated steatohepatitis. Cell Metab.

[42] Masoodi M, Gastaldelli A, Hyötyläinen T (2021). Metabolomics and lipidomics in NAFLD: biomarkers and non-invasive diagnostic tests. Nat Rev Gastroenterol Hepatol.

